# Repair of a Strip Perforation with Calcium-Enriched Mixture Cement: A Case Report

**Published:** 2014-07-05

**Authors:** Mohammad Jafar Eghbal, Mahta Fazlyab, Saeed Asgary

**Affiliations:** a Dental Research Center, Research Institute of Dental sciences, Shahid Beheshti University of Medical Sciences, Tehran, Iran; b Iranian Center for Endodontic Research, Research Institute of Dental Sciences, Shahid Beheshti University of Medical Sciences, Tehran, Iran

**Keywords:** Calcium-Enriched Mixture Cement, CEM Cement, Endodontics, Furcation Defects, Perforation Repair, Root Canal Therapy, Root Perforation

## Abstract

The present report reviews the diagnostic and treatment challenges of a mandibular molar with previous root canal treatment and signs of a procedural mishap, *i.e.* furcal radiolucency and localized swelling of the gingival margin in which a sinus tract was present. By tracing the sinus tract, it became evident that the lesion originated from the furcation area, not the root apices. This case was treated by cleaning/filling the coronal half of the canals and leaving the rest of obturating material untouched. The strip perforation zone in the mesial root was sealed off with calcium-enriched mixture cement. One week after treatment, the patient’s symptoms had faded away and one year later, the lesion completely healed with bone replacement.

## Introduction

Even though a root canal filling might conform to the state-of-the-art in science and technology, the possibility of failure cannot be excluded as microbial leakage can compromise the success of nonsurgical root canal therapy, and the quality of the coronal seal is just as important as the technical quality of the root canal filling for periapical health after root canal therapy (RCT) [1].

Apart from microbial debridement of the root canal system, the success of root canal therapy is based on achieving seal. The basis for this belief came from Hunter’s focal infection theory [2], Rosenow’s concept of elective localization [3], and the hollow-tube theory by Rickert and Dixon [4] stating that those bacteria which have survived the chemo-mechanical debridement of the root canal system or that persisted within the remaining filling materials, are capable of inducing endodontic failure. The presence of bacteria is known as the main reason for failure of endodontic treatment [5].

Accidental root perforation may also complicate the endodontic treatment *per se* [6-9]. Perforation can be defined as a pathologic/artificial communication between the root canal system and the external tooth surface [9, 10]. Mesiobuccal roots of maxillary molars and the mesial roots of mandibular molars are highly susceptible to strip perforation because of thin dentinal walls. Inappropriate instrumentation not avoiding the danger zone and over preparation of these thin root canals can cause strip perforation [8]. Extended defects and longtime elapse before repair, are accompanied by poorer prognosis due to down-growth of gingival epithelium just below the perforation site, especially when accidental perforations occur in the crestal area of two- and multi-rooted teeth [6, 11]. Bacterial infection originating from the root canal and/or periodontal tissues, results in inflammatory processes accompanied by pain and tenderness, suppuration, abscesses, and sinus tracts [7, 11]. For diagnostic purposes, it is essential for the clinician to trace the lesion by inserting a gutta-percha cone into the sinus tract and to take one or more radiographs to determine the origin of the lesion [12].

Successful management of root perforation depends on early diagnosis of the defect, choice of treatment and materials, host response, and the experience of the practitioner [10]. The orthograde treatment of root perforation follows the same rational of conservative endodontic therapy, *i.e.* prevention and treatment of periradicular inflammation [11]. This may be achieved by controlling the infection of the perforation site, or if already infected, by using procedures that can disinfect the area and provide the best possible seal against penetration of bacteria and their byproducts [6, 11].

The ideal material for perforation repair should be antibacterial, radiopaque, non-cytotoxic, non-absorbable, biocompatible and able to induce formation of hard tissue, particularly cementum, over the material and provide a three-dimensional seal [13-15]. Calcium-enriched mixture (CEM) cement was introduced as a hydrophilic tooth-colored biomaterial with favorable sealing ability. CEM is biocompatible, nontoxic for the pulp, and antibacterial. It is also proved to be hard tissue inductive; dentinogenic, cementogenic and osteogenic [16]. All these properties make CEM a valid biomaterial for cases of perforation repair.

This case report has focused on the diagnostic and treatment challenges of a furcation perforation in a mandibular molar; also, the one-year post-operative successful treatment outcomes are represented.

## Case Report

**Figure 1:**
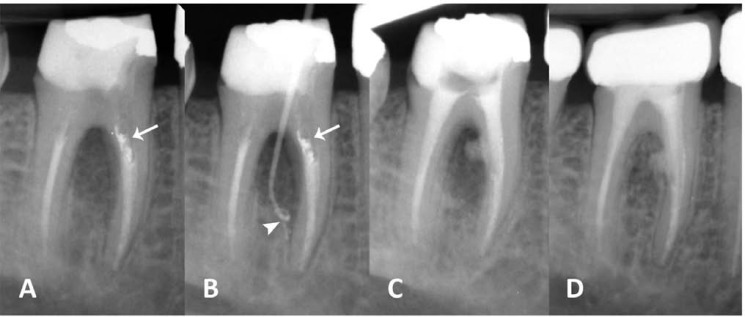
*A) *Pre-operative parallel radiography of the lower first molar with an extensive furcation lesion and defective coronal restoration. Note the opaque material in the mesial root (white arrow); *B)* Diagnostic radiography after tracing the sinus tract with a gutta-percha cone that ends up within the furcal lesion (white arrow head). Again, note the opaque cement in the mesial root (white arrow); *C)* Post-operative radiograph; note the flow of CEM cement into the furcation area. The apical 1/2 of all canals are left untouched; *D) *One year follow-up radiography shows complete healing of the furcal lesion and its replacement with bone

A healthy 35-year-old male with no medical complication resorted, complaining of a dull pain in the mandibular first molar area on the left side. He stated that RCT of the aforementioned tooth was done by a general practitioner one year earlier. Upon clinical examination the tooth contour showed that it had been prepared for prosthetic crown which according to the patient was later removed to help in eliminating the tooth abscess. The tooth had a defective discolored composite build-up and was not mobile or tender to percussion. Visual scanning revealed a local tender inflammation overlying the buccal mucosa in the furcal region. A draining sinus tract was evident ~2 mm from the gingival margin within the keratinized mucosa. Careful periodontal probing of the tooth showed that pocket depth was within the normal range (<3mm). On a parallel radiography, previous RCT had a moderate quality. A large inter-radicular lesion was evident (Figure 1A). The sinus tract was traced with a #30 gutta-percha point (Ariadent, Tehran, Iran) and according to the second radiograph, its path did not lead to the root apices indicating that the lesion was not related to the apical and middle zones of the root canals (Figure 1B). On both cliché, an opaque bulk of material was evident on the coronal section of the mesial root filling that suggested the existence of an unusual event (*i.e.* strip perforation) and dentist’s effort to seal off that area which could potentially be the source of lesion (Figure 1A and 1B).

The possible treatment options including tooth extraction with/without replacement and perforation repair with orthograde re-accession and coronal restoration were explained for the patient. In accordance with the patient preferences, the option of saving the tooth via strip perforation repair with CEM cement was chosen. The patient signed an informed consent.

After administering 0.2% chlorhexidine rinse (Behsa Co., Tehran, Iran), the tooth was isolated. The restorative material was removed and all canal orifices were located. The coronal ~4-5 mm of the root filling material was extirpated and 5.25% NaOCl was left in the canals for ~5 minutes. Meanwhile CEM cement (BioniqueDent, Tehran, Iran) was prepared according to manufacturer's instruction. After drying the canals with paper points (Ariadent, Tehran, Iran), CEM cement was placed into the orifices. The biomaterial was gently packed with a dry cotton pellet and appropriate paper points to obtain a good adaptation. Then it was covered with a moistened cotton pellet and the tooth was temporarily restored (Coltosol; AsiaChemiTeb Co., Tehran, Iran). A control post-operative radiography showed the flow of CEM filling through the perforation site into the lesion that confirmed the pre-operative diagnosis (Figure 1C).

On a subsequent visit one week later, all sings/symptoms had subsided and the buccal swelling in the gingiva had faded away. The patient was referred for the prosthetic treatment of the tooth. One-year follow-up radiography revealed complete healing of the lesion and its replacement with bone (Figure 1D). The tooth was totally functional and symptomless.

## Discussion

This article represented the diagnostic and treatment challenges of a previously perforated mandibular molar with periodontal abscess that was successfully treated with CEM cement.

Primary endodontic disease with secondary periodontal involvement, primary periodontal disease with secondary endodontic involvement, or true combined diseases are clinically and radiographically very similar [12, 17, 18]. In this case, the furcation abscess could have been mistakenly diagnosed as a primary periodontal lesion. As no sign of periodontitis or even gingivitis was present, this differential diagnosis was ruled out.

The disease associated with radiolucency around endodontically treated teeth is apical periodontitis which may have persisted despite treatment, reoccurred after initial healing, or emerged during the post-operative follow-up period, so it’s appropriate to characterize it as post-treatment disease (PTD) [19, 20]. PTD, like other disease processes, can be resolved only if the etiological factor is eliminated or effectively curtailed. As mentioned earlier, the cause of PTD in this current case was not the quality of previous apical seal; despite being unsuccessful, there was an attempt to seal off the strip perforation in the mesial root with an opaque cement (Figure 1A and 1B). It is obvious that not only the endodontic retreatment of the tooth wouldn’t eliminate the cause of the disease but also it could potentially worsen the situation by enlarging the perforation during the action of instruments and intensifying the obturation. In other words, endodontic retreatment would not change the outcome and repair of the perforation site remained essential. Complete healing of the furcation lesion after one year confirmed this decision (Figure 1D).

Another issue is the importance of coronal seal which appears to be of equal, if not greater, clinical relevance compared to apical leakage as a cause of endodontic failure [21]. Coronal leakage can occur along the restoration margins through the endodontic filling. According to Ray and Trope, defective restorations and adequate root canal fillings have a higher incidence of failures than teeth with inadequate root canal fillings and adequate restorations. In some studies the influence of coronal seal on periapical status is stated to be much more than that of a well qualified RCT [22]. From all these data, it can be assumed that co-existence of coronal leakage and accidental endodontic periodontal communication pathway had caused the problem and a hermetic seal could resolve it. 

CEM cement was introduced as an endodontic filling material. This cement has favorable properties such as flow, film thickness, antimicrobial properties, and biocompatibility [10, 16, 23-26]. Creation of a three-dimensional seal is highly important in the success of perforation repair [11]. The sealing ability of CEM which improves in the presence of phosphate-buffered solution (PBS), is comparable to MTA [9], and ensures the perfect outcome of the treatment. In addition, the formation of bone in the healed lesion cannot be overlooked. Hard-tissue (bone, cementum and dentin) inducing ability of CEM is proved in many studies [14, 27]. This phenomenon can be due to its sealing ability, biocompatibility, high alkalinity, and antibacterial effect [27]. Moreover, CEM has the ability to promote hydroxyapatite formation which can be another reason for its hard tissue inducing property [16].

## Conclusion

Smart combination of correctly chosen treatment and material and correct diagnosis of the etiology, is the key to successful treatment. As a biocompatible hard tissue inducing material, CEM cement may effectively be used for repair of procedural perforations.
